# Modulators of Energy Expenditure Accuracy in Adults with Overweight or Obesity: E-MECHANIC Secondary Analyses

**DOI:** 10.1249/MSS.0000000000003583

**Published:** 2024-10-15

**Authors:** RACHEL MATTHEWS, CHRISTOPH HÖCHSMANN, MELISSA L. ERICKSON, JAMES L. DORLING, GUILLAUME SPIELMANN, NEIL M. JOHANNSEN, TIMOTHY S. CHURCH, CORBY K. MARTIN

**Affiliations:** 1 School of Kinesiology, College of Human Sciences & Education, Louisiana State University, Baton Rouge, LA; 2 Department of Health and Sport Sciences, TUM School of Medicine and Health, Technical University of Munich, Munich, GERMANY; 3 Translational Research Institute, AdventHealth, Orlando, FL; 4 Human Nutrition, School of Medicine, Dentistry and Nursing, College of Medical, Veterinary and Life Sciences, University of Glasgow, UNITED KINGDOM; 5 Pennington Biomedical Research Center, Baton Rouge, LA

**Keywords:** EXERCISE INTERVENTION, METABOLIC EQUATIONS, OFFSET FACTOR

## Abstract

**Purpose:**

American College of Sports Medicine (ACSM) metabolic equations are used to estimate energy expenditure (EE) of physical activity and prescribe aerobic exercise to meet EE requirements. Limited evidence supports their accuracy in sedentary adults with overweight or obesity during controlled exercise interventions. The purpose of this study was to compare EE estimated by the ACSM walking equation versus EE measured by indirect calorimetry during a 24-wk aerobic exercise intervention, and identify potential modulators for their accuracy.

**Methods:**

Data from the exercising groups (8 or 20 kcal·kg body weight^−1^·wk^−1^) of the E-MECHANIC study were utilized in this ancillary analysis (*N* = 103). Every 2 wk for the initial 8 wk and monthly thereafter, EE was measured via indirect calorimetry during absolute (2 mph, 0% grade) and relative (65%–85% peak oxygen uptake (V̇O_2peak_)) workload exercise. Resting metabolic rate, V̇O_2peak_, and body composition were assessed at baseline and follow-up. An EE offset factor (EOF) was calculated to express measured EE as a percentage of the estimated EE at each workload (EOF < 100% represents an overestimation of ACSM estimated EE).

**Results:**

The accuracy of the equation decreased with increasing exercise workload (0.44%, 9.2%, and 20.3% overestimation at absolute, relative, and maximal workloads, respectively, at baseline) and overestimation of EE was greater after the exercise intervention. Furthermore, race, sex, age, fat mass, and V̇O_2peak_ were identified as modulators for equation accuracy. Greater overestimation of EE was observed in Black compared with White females, particularly at lower exercise workloads.

**Conclusions:**

These findings support future efforts to improve the accuracy of metabolic equations, especially in diverse populations. Researchers should account for exercise efficiency adaptations when using metabolic equations to prescribe exercise precisely.

## INTRODUCTION

With increasing prevalence of obesity (1,2), intervention strategies to facilitate weight management are becoming increasingly important. Among these strategies, aerobic exercise is popular for its role in weight loss and associated health benefits (3–5). Most simply, aerobic exercise increases daily energy expenditure (EE) and facilitates weight maintenance or aids weight loss when combined with an isocaloric or hypocaloric diet. Methods for estimating physical activity EE can be useful in setting and meeting caloric goals or weight loss targets.

Metabolic equations provide an accessible way for researchers, practitioners, and fitness professionals to estimate EE and prescribe aerobic exercise to clients based on goals (e.g., general health or weight loss) and preferred exercise mode, intensity, duration, and frequency. The American College of Sports Medicine (ACSM) developed metabolic equations to estimate gross EE during various physical activities, including walking and running (6). These metabolic equations have resting, horizontal, and vertical components to estimate oxygen consumption, and ACSM guidelines include conversions to EE using body weight and assumptions regarding substrate oxidation (7). In the case of treadmill exercise, speed and grade are required to calculate the horizontal and vertical work components, respectively. Several studies, recruiting a range of participants, have used the ACSM metabolic equations to estimate gross EE during exercise tests and interventions (8–11). However, the evidence used to develop the ACSM metabolic equations was collected in a small sample (*n* = 3 and *n* = 2, respectively) of young and trained males with a healthy body weight and either Caucasian (12) or of unknown race (13); therefore, the accuracy of the metabolic equations may be impaired in more diverse populations, including females and individuals with greater body weights or adiposity.

Despite consistent health benefits, heterogeneity in body weight changes in response to aerobic exercise has been identified (14). As part of this heterogeneity, there are individuals who experience less weight loss (e.g., measured pre- vs post-exercise intervention) than expected (e.g., body weight loss that is estimated by metabolic equations) and have been characterized as “weight compensators.” The explanation for this body weight compensation may be due to several factors, such as metabolic and/or behavioral adaptations that offset increases in exercise EE and therefore reduce the amount of body weight loss (15). However, another explanation for body weight compensation may be related to imprecise energy balance prescriptions. For example, ACSM metabolic equations have been found to overestimate EE, particularly in populations different from the sample used to develop the metabolic equations, including coronary artery disease patients and females with healthy weight and overweight (16–18). Therefore, it is plausible that inaccurate exercise prescriptions using the ACSM metabolic equations are partially responsible for attenuated weight loss. In individuals with excess adiposity, the ACSM metabolic equations may be particularly inaccurate due to the lower metabolic activity of adipose tissue relative to skeletal muscle (19). Moreover, the effect of adiposity may be compounded by known race and sex differences in metabolism and body composition (20,21), and demographic and anthropometric variables may partially account for variation in the inaccuracy of the existing metabolic equations.

The present study aimed to evaluate the accuracy of the ACSM metabolic equation for walking by comparing equation-derived estimates of EE to measured EE quantified by indirect calorimetry in sedentary, White and Black adults with overweight or obesity undergoing a 24-wk aerobic exercise intervention. Furthermore, we aimed to identify potential anthropometric and demographic modulators of EE estimation accuracy. Exercise training status may be another factor that impacts EE estimation accuracy, and the initial participant sample used to derive the metabolic equations consisted of trained males (12,13). Therefore, we hypothesized that EE estimation accuracy would improve over the course of the exercise intervention as participants became more trained. In addition, given the previous findings on sex- and race-specific differences in energy metabolism and adiposity (20,21), we hypothesized reduced accuracy of EE estimation in females and Black participants compared with White males and greater overestimation of EE in individuals with higher body fat percent.

## METHODS

### Study design

The present work is a secondary analysis of previously collected data from the Examination of Mechanisms of Exercise-Induced Weight Compensation (E-MECHANIC) study. The parent study consisted of a three-arm, 24-wk randomized controlled trial (22–24). Herein, we considered data from the two exercise conditions and did not include data from the third (control) arm in the analysis. Specifically for the present analysis, we compared estimated versus measured EE before, during, and after the exercise intervention. In E-MECHANIC, participants were randomly assigned to an exercise dose designed to reflect recommendations for either general health (8 kcal·kg body weight^−1^·wk^−1^ (KKW)) or weight loss (20 KKW) (25). During the intervention, participants underwent a 24-wk supervised treadmill exercise training program at the prescribed dose, conducted at Pennington Biomedical Research Center (Baton Rouge, LA). Resting metabolic rate (RMR), peak oxygen uptake (V̇O_2peak_), and body composition (assessed via dual-energy x-ray absorptiometry (Lunar iDXA with Encore software version 13.60; GE Healthcare, Madison, WI)) were measured before and after completion of the 24-wk exercise intervention.

### Participants

Of 133 exercising participants in the E-MECHANIC study, 103 were included in the present analysis. The 30 participants not included in the present analysis were excluded for having less than 70% compliance (attendance at exercise sessions) and/or not completing baseline and follow-up assessments. Participants had overweight or obesity (body mass index (BMI) ≥25 and ≤45 kg·m^−2^) and were sedentary (not exercising more than 20 min, 3 d·wk^−1^), as assessed by self-report and 1-wk accelerometer data. Exclusion criteria included drinking more than 14 alcoholic drinks per week, smoking in the last 6 months, pregnancy or having been pregnant within the last 6 months, breastfeeding, previous weight loss surgery, current participation in a weight loss program, medical conditions (e.g., cardiovascular disease or diabetes), and inability to safely complete the prescribed exercise dose. The Institutional Review Board of Pennington Biomedical Research Center approved the E-MECHANIC study, and complete trial details have been previously published (22).

### Exercise intervention protocol

In both the 8 KKW and 20 KKW groups, participants exercised on a treadmill (LifeFitness, Rosemont, IL) at a self-selected workload within a heart rate range of 65%–85% of baseline V̇O_2peak_ on 3–5 d·wk^−1^ for 24 wk. Caloric expenditure goals for each exercise session were calculated by dividing the prescribed exercise volume by session frequency (3–5 d·wk^−1^, depending on participant preference). EE was calculated in real time during each training session using speed, grade, body weight, and the ACSM metabolic equation for walking (6). The estimated EE information allowed for the adjustment of session duration to meet participants’ caloric expenditure goals. During the first week of the intervention, every 2 wk for the initial 8 wk, and monthly thereafter, EE was measured at an absolute (2 mph, 0% grade) and relative workload (current speed and grade to meet prescribed workload) using the ParvoMedics True Max 2400 Metabolic Cart (Sandy, UT) as part of a 12-min intervention quality control protocol designed to ensure accurate estimation of EE throughout the exercise intervention. Following 2 min of rest, participants exercised for 5 min at each workload, and oxygen consumption during the last 2–3 min of each 5-min period was used in subsequent analyses to ensure values reflected steady state exercise. A correction factor, calculated by dividing the measured EE collected during the 12-min quality control protocol by the ACSM estimated EE, was applied to real-time EE calculations during subsequent exercise sessions. The correction factor was designed to enable more accurate adherence to the prescribed exercise dose, given that our study population differed from the original population used to develop the ACSM metabolic equations and the hypothesis that metabolic equations may not precisely estimate actual EE. The correction factor further allowed us to adjust the exercise duration to account for changes in metabolic or biomechanical efficiency and to ensure calories carried over from previous weeks remained equivalent throughout the study. Estimated EE values included in the analyses reflect values calculated from the ACSM metabolic equations and do not include the correction factor, which was applied to ensure accurate compliance to the prescribed exercise dose. Moreover, a quality control check, to ensure accurate speed and grade across the entire capacity of the treadmill used for the exercise intervention, was conducted once a month using a digital tachometer and inclinometer.

### Resting metabolic rate

Indirect calorimetry was used to assess RMR following a 12-h overnight fast. Participants rested in a supine position for 30 min in a quiet room with the lights dimmed. During this time, a ventilated hood was placed over the participants head and shoulders, which was connected to a MAX-II metabolic cart system (AEI Technologies, Pittsburgh, PA) to continuously analyze expired gases. Of the 30-min assessment, data collected from the last 20 min were averaged and reported as measured resting EE (22).

### V̇O_2peak_ testing

A standardized graded walking treadmill exercise test was used to assess V̇O_2peak_ before and after the exercise intervention. For the first stage, the treadmill (Trackmaster 425, Newton, KS) was set to 2.4 mph and 0% grade. Every 2 min, speed and/or grade was increased following a standardized protocol to induce a 1 metabolic equivalent of task (MET) increase until volitional fatigue. Respiratory gases were analyzed throughout the test using the ParvoMedics True Max 2400 Metabolic Cart (Sandy, UT). Heart rate and Borg’s scale for ratings of perceived exertion (26) were also monitored at the end of each stage.

### EE calculations

The following ACSM metabolic equation was used to calculate gross EE during walking, with each term of the equation representing the resting, horizontal, and vertical components, respectively (6).


$$\text{Walking}:\dot{V}O_{2}\;\left(\mathrm{mL}\cdot {\mathrm{kg}}^{-1}\cdot \min^{-1}\right)=3.5+\left(0.1\times \text{speed}\right)+\left(1.8\times \text{speed}\times \text{grade}\right)$$

Estimation of resting EE was calculated using 3.5 mL·kg^−1^·min^−1^, in accordance with the resting component of the ACSM metabolic equations. Estimated oxygen consumption (V̇O_2_; mL·kg^−1^·min^−1^) was converted to EE (kcal·min^−1^) using the assumption that 1 L of oxygen equals approximately 5 kcal of EE (7).

An EE offset factor (EOF) was calculated to represent the accuracy of the metabolic equation by expressing measured EE as a percent of the ACSM equation estimated EE. An EOF of 100% represents complete agreement between the measured and estimated EE. An EOF of less than 100% indicates an overestimation of EE by the ACSM metabolic equation. Conversely, an EOF of greater than 100% indicates an underestimation of EE by the ACSM metabolic equation.

Data presented in this study largely reflect gross EE unless otherwise specified. Net EE was calculated by removing the resting components from measured and estimated EE. Specifically, measured net EE was calculated by subtracting measured resting EE from the measured exercising gross EE. Estimated net EE was calculated by subtracting the resting component of the metabolic equation (3.5 mL·kg^−1^·min^−1^) from the estimated exercising gross EE. Net EOF was calculated using measured net and estimated net EE to represent the accuracy of the metabolic equation after adjusting for RMR.

### Statistical Analysis

Statistical analysis was conducted using JMP (Version 17.0.0; SAS Institute Inc., Cary, NC). One-way ANOVA was used to compare differences in baseline and follow-up characteristics between exercise doses. Two-way repeated-measures ANOVA was used to test for the effect of time, estimated versus measured EE, and time–estimated vs measured EE interaction on resting, absolute, relative, and maximal EE. Subject ID, subject ID–time, and subject ID–estimated versus measured EE were entered into the model as random factors. Correlations between estimated and measured EE at each workload were calculated using a general linear model, with estimated EE, time, and estimated EE–time interaction entered as fixed effects. Because of the small sample of Black males in this study (Table 1), the combined effects of race and sex were analyzed using a categorical variable (RACE&SEX) with four categories (White males, Black males, White females, Black females). Two-way repeated-measures ANOVA was used to test for the effect of RACE&SEX, time, and RACE&SEX–time interaction on estimated EE, measured EE, oxygen consumption (V̇O_2_), respiratory exchange ratio (RER), and EOF at an absolute and relative workload, which was collected at baseline, follow-up, and intermittently throughout the study as detailed above. Subject ID-by-week and subject ID nested by RACE&SEX (ID[RACE&SEX]) were included as random effects. ANOVA was also used to test for the effect of RMR adjustment (i.e., gross vs net EE), RACE&SEX, time, and time-by-RMR adjustment interaction on EOF at absolute, relative, and maximal workload. Subject ID, ID-by-time, and ID-by-RMR adjustment were included as random effects. All *post-hoc* pairwise comparison for interactions were conducted using Student’s *t*-tests. Statistical significance was set at *P* < 0.05 for all analyses.

**TABLE 1:** Baseline participant characteristics.

	White Males	White Females	Black Males	Black Females	Total
*n*	26	46	4	27	103
Group, *n* (% 20 KKW)	11 (42)	25 (54)	3 (75)	9 (33)	48 (47)
Age (y)	48.9 (14.7)	50.0 (10.8)	56.3 (6.9)	45.0 (10.3)	48.7 (11.8)
Weight (kg)	98.4 (16.7)	79.2 (10.6)	94.7 (12.6)	92.1 (12.3)	88.0 (15.2)
BMI (kg·m^−2^)	31.1 (4.8)	29.3 (3.6)	31.3 (4.8)	33.6 (4.5)	31.0 (4.5)
Lean mass (kg)	59.9 (8.2)	40.8 (3.8)	62.9 (9.7)	46.7 (4.1)	48.0 (10.0)
Body fat (%)	35.2 (5.0)	44.6 (4.7)	29.2 (8.0)	45.2 (5.5)	41.8 (7.0)
RMR (kcal·d^−1^)	1812 (242)	1378 (206)	1835 (337)	1437 (153)	1521 (280)
$\dot{V}$O_2peak_ (mL·kg^−1^·min^−1^)	28.0 (5.4)	23.1 (4.7)	24.3 (8.0)	20.8 (3.3)	23.8 (5.3)

Data are mean (SD).

Stepwise multiple regression was used to predict baseline EOF at each workload by the following seven independent variables measured at baseline: age, RACE&SEX, RMR, V̇O_2peak_, fat mass, lean mass, and body fat percent, with the exception of RMR for resting EOF and V̇O_2peak_ for max EOF because these variables were directly used in the EOF calculations. In addition, stepwise multiple regression was used to predict changes in EOF from baseline to follow-up at each workload, with the same seven independent variables used, but calculated as change values (where appropriate) from baseline to follow-up. A *P* value threshold of 0.25 to enter and 0.1 to leave the stepwise regression was used, and significance was set at *P* < 0.05 for the final model. In order to address possible multicollinearity violations, variance inflation factor (VIF) for each independent variable was calculated for every model. VIF did not exceed 5 in any of the models; therefore, we can assume that multicollinearity did not influence the models produced by the stepwise regression. Part *r* was also calculated by square root of the quotient of the type III sum of squares divided by the total sum of squares.

## RESULTS

### Participant Characteristics

A total of 103 exercising participants (71% female, 70% White) completed the 24-wk exercise intervention with at least 70% compliance (Table 1). White females represented 45% of the sample, whereas Black males accounted for 4% of participants. Age ranged from 21 to 65 yr (48.7 ± 11.8 yr).

#### Changes in EE at rest and absolute and relative workload exercise across the course of the intervention were not different between 8 KKW and 20 KKW doses. Therefore, exercise groups were combined for subsequent data analysis

The exercise groups (8 KKW and 20 KKW) were analyzed for potential cross-sectional differences at baseline and follow-up (week 24) and for longitudinal changes between baseline and follow-up (Table 2). Body weight, lean mass, and percent body fat did not differ between exercise groups at baseline or follow-up (*P* ≥ 0.19 for all). However, the 20 KKW group showed greater weight and percent body fat losses than the 8 KKW group over the course of the intervention (*P* ≤ 0.02). Baseline and follow-up values for V̇O_2peak_ were not different between the exercise groups (*P* = 0.75 and *P* = 0.11, respectively), but improvements in V̇O_2peak_ were greater in those randomized to the 20 KKW intervention compared with 8 KKW (3.3 ± 3.1 vs 0.9 ± 2.6 mL⋅kg^−1^⋅min^−1^, *P* < 0.01). Baseline, follow-up, and change values for RMR and absolute and relative EE were not different between the exercise doses (*P* ≥ 0.09 for all). Maximum EE during the V̇VO_2peak_ test was not different at baseline or follow-up (*P* ≥ 0.38 for both); however, the increase from baseline to follow-up was greater in the 20 KKW compared with 8 KKW group (1.35 ± 1.05 vs 0.42 ± 1.14 kcal⋅min^−1^, *P* < 0.01).

**TABLE 2:** Comparison between the intervention groups at baseline and follow-up, and the change from baseline to follow-up.

		Baseline			Follow-up			Change	
	8 KKW	20 KKW	*P*	8 KKW	20 KKW	*P*	8 KKW	20 KKW	*P*
Weight (kg)	89.4 (15.6)	86.5 (14.7)	0.33	88.3 (16.0)	84.3 (14.6)	0.19	−1.1 (2.4)	−2.2 (2.6)	0.02*
Lean mass (kg)	48.9 (9.8)	47.0 (10.1)	0.35	48.6 (10.1)	47.0 (10.0)	0.41	−0.2 (1.2)	−0.2 (1.0)	>0.99
Body fat (%)	41.7 (6.6)	41.9 (7.6)	0.85	41.5 (6.6)	40.9 (7.5)	0.66	−0.1 (1.3)	−0.9 (1.7)	0.01*
V̇O_2peak_ (mL·kg^−1^·min^−1^)	23.9 (5.5)	23.6 (5.1)	0.75	24.9 (6.3)	26.9 (6.3)	0.11	0.9 (2.6)	3.3 (3.1)	<0.01*
RMR (kcal·d^−1^)	1539 (261)	1500 (303)	0.49	1589 (349)	1528 (350)	0.38	50 (219)	28 (204)	0.59
Absolute EE (kcal·min^−1^)	3.92 (0.79)	3.83 (0.68)	0.55	3.68 (0.66)	3.62 (0.68)	0.67	−0.24 (0.36)	−0.21 (0.41)	0.86
Relative EE (kcal·min^−1^)	6.50 (1.26)	6.28 (1.25)	0.38	8.26 (1.85)	8.51 (2.52)	0.58	1.76 (1.14)	2.22 (1.91)	0.09
Max EE (kcal·min^−1^)	10.56 (2.69)	10.11 (2.70)	0.40	10.98 (2.95)	11.50 (3.05)	0.38	0.42 (1.14)	1.35 (1.05)	<0.01*

Data are mean (SD). Absolute, treadmill exercise at 2 mph and 0% grade; Relative, current speed and grade to meet prescribed training workload of 65%–85% of baseline V̇O_2_peak; Max, highest values achieved during the V̇O_2peak_ test. 8 KKW, *n* = 55; 20 KKW, *n* = 48.

Because the changes in EE at rest, absolute, and relative workload were not significantly different between the exercise groups, further analysis combined the 8 KKW and 20 KKW data.

#### In healthy individuals with overweight/obesity, EE is overestimated by the metabolic equation at rest and during exercise, and an exercise intervention may increase exercise efficiency at low workloads

**FIGURE 1:**
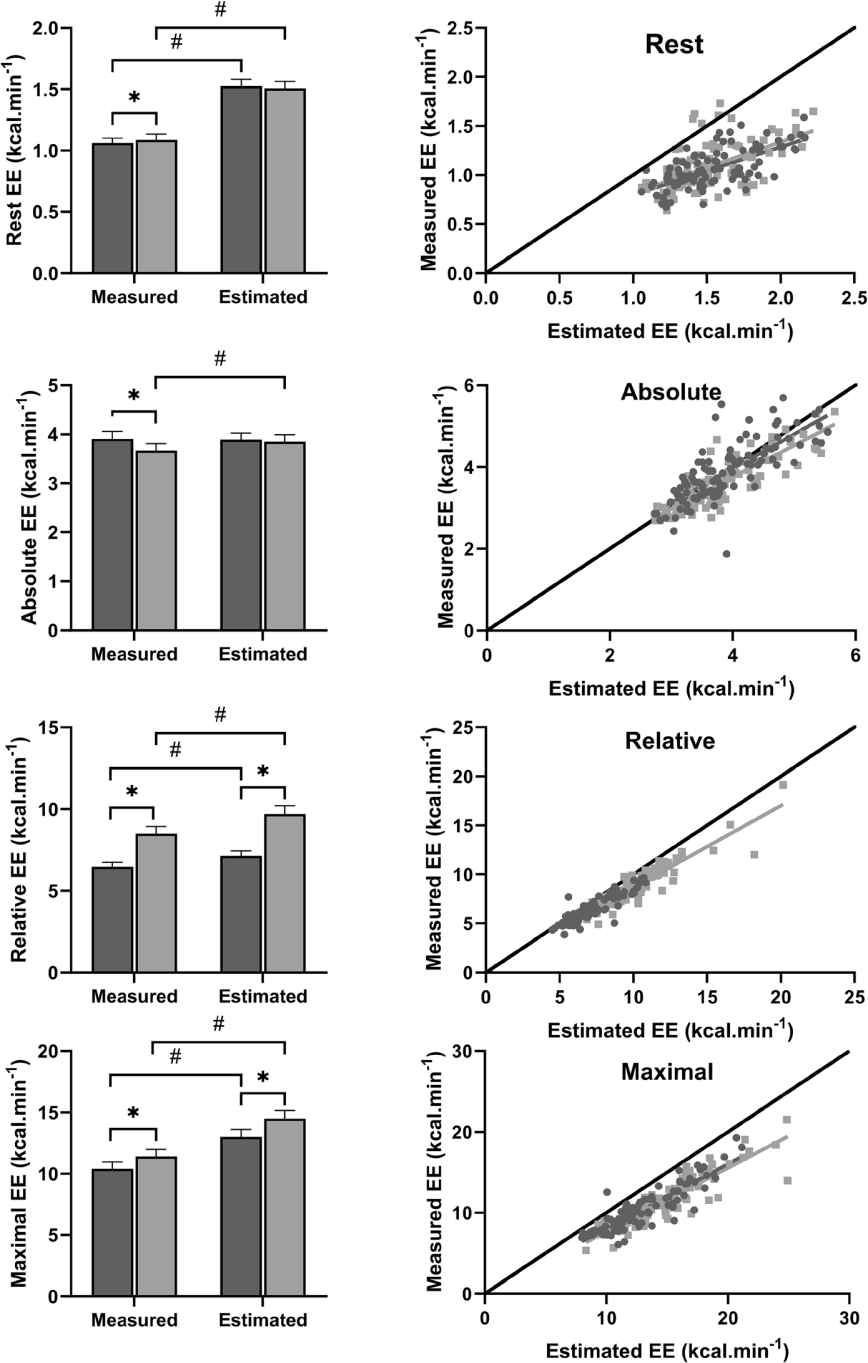
Measured and estimated EE at rest, absolute workload (2.0 mph, 0% grade), relative workload (65%–85% V̇O_2peak_), and maximal workload (maximum EE achieved during graded V̇O_2peak_ test). Measured EE collected from indirect calorimetry and estimated EE calculated using the ACSM’s equation for treadmill walking. ● baseline; ● follow-up. *Significantly different from baseline, *P* < 0.05. #Significantly different from estimated EE within a time point, *P* < 0.05. Bar chart presents mean with error bars indicating SD.

At rest, measured EE was 30.4% and 27.9% lower than the estimated EE at baseline and follow-up, respectively (Fig. 1; *P* < 0.01 for both). The ACSM metabolic equation most accurately estimated EE during the absolute (lowest) workload exercise at baseline (0.44% difference; *P* = 0.71); however, after the intervention, the discrepancy between measured and estimated EE increased, with the metabolic equation overestimating EE by 5.1% (*P* < 0.01). The percent difference between estimated and measured EE increased with increasing exercise workloads; exercise at a relative workload exhibited greater percent differences than at an absolute workload, and maximal exercise showed even greater discrepancies. Relative workload exercise displayed 9.2% and 12.6% differences in measured and estimated EE at baseline and follow-up, respectively, compared with 20.3% and 21.5% during maximal exercise (*P* < 0.01 for baseline and follow-up at relative and maximal workloads). At rest, a moderate correlation exists between estimated and measured EE (*r* = 0.67). During exercise, a strong correlation exists between estimated and measured EE at all exercise workloads (*r* ≥ 0.79 for all).

Over the course of the 24-wk intervention, measured resting EE increased by 2.4% (*P* = 0.02). Measured EE at an absolute workload decreased by 6.2% from baseline to follow-up (*P* < 0.01). During relative workload exercise, measured EE increased by 30.9% (*P* < 0.01); however, this was accompanied by a 0.4 mph and 2.9% increase in speed and grade, respectively. Similarly, maximum EE during the V̇O_2peak_ test increased by 9.4% from baseline to follow-up, in accordance with a 0.1 mph and 2.1% mean increase in maximal speed and grade, respectively.

#### Age, race and sex, cardiorespiratory fitness, and anthropometric variables significantly predicted variance in baseline EOF at varying workloads, but changes in these modulators from baseline to follow-up were not related to changes in EOF

A stepwise regression was used to determine which demographic and anthropometric variables were associated with baseline EOF at each workload and, therefore, could contribute toward the variance in estimation equation accuracy. Variance in resting EOF was significantly predicted by fat mass (part-*r* = 0.319, *P* < 0.001), V̇O_2peak_ (part-*r* = 0.270, *P* < 0.001), and RACE&SEX (part-*r* = 0.139, *P* = 0.05). Absolute EOF was also explained by fat mass (part-*r* = 0.225, *P* = 0.01) and RACE&SEX (part-*r* = 0.272, *P* = 0.003). Furthermore, variance in EOF at a relative workload was predicted by fat mass (part-*r* = 0.241, *P* = 0.01). Finally, maximal EOF was partially explained by age (part-*r* = 0.405, *P* < 0.001), RACE&SEX (part-*r* = 0.226, *P* = 0.01), and fat mass (part-*r* = 0.219, *P* = 0.01).

Stepwise regression was also used to identify potential modulators for changes in EOF values at each workload. A significant amount of variability for change in resting EOF was explained by V̇O_2peak_ (part-*r* = 0.422, *P* < 0.001), but variability in other workloads was not significantly explained by the variables entered into the model.

#### A 24-wk exercise intervention elicited metabolic adaptations to exercise (assessed via measured EE, RER, V̇O_2_, and EOF) within the first 8 wk, with additional sex and race effects for these metabolic measurements

**FIGURE 2:**
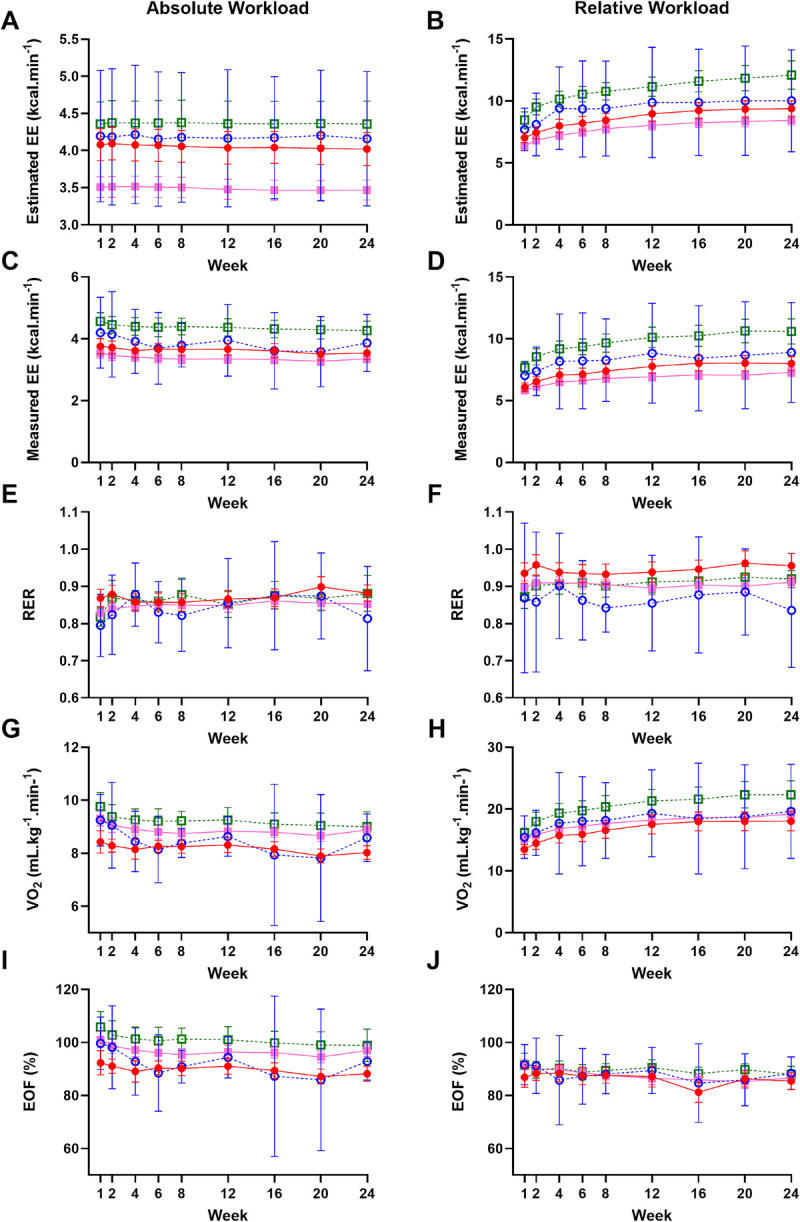
Changes in estimated (A and B) and measured (C and D) EE, RER (E and F), V̇O_2_ (G and H), and EOF (I and J) during absolute (2.0 mph, 0% grade) and relative workload (65%–85% V̇O_2peak_) exercise across the 24-wk intervention. Measured EE collected via indirect calorimetry. Estimated EE calculated using the ACSM’s metabolic equation for treadmill walking. Black females; White females. Solid lines represent females; dotted lines represent males. Data presented as means with error bars indicating 95% confidence interval.

Changes in estimated and measured EE, RER, V̇O_2_, and EOF during absolute and relative workload exercise were tracked over the course of the 24-wk exercise intervention (Fig. 2). RACE&SEX effects were found for all measures across both absolute and relative workloads (*P* < 0.01 for all), except RER at an absolute workload (*P* = 0.24) and EOF at a relative workload (*P* = 0.43).

Estimated EE at an absolute workload did not change over the course of the intervention due to the fixed treadmill speed and grade and modest weight changes (Fig. 2A). However, at a relative workload, estimated EE increased over the course of the intervention, reflective of increases in speed and/or grade (*P* < 0.01), with increases between weeks 1 to 2, 2 to 4, 4 to 8, and 8 to 12 (Fig. 2B). From week 16 onward, there was no additional increase in estimated EE. A RACE&SEX–time interaction also existed for estimated EE at a relative workload (*P* < 0.01), with White males displaying higher EE than White females and Black females at all weeks. Furthermore, Black females had higher estimated EE than White females at week 4 and onward from week 12.

An effect of time existed at both absolute and relative workloads for measured EE (*P* < 0.01 for both). Measured EE at an absolute workload was higher in weeks 1 and 2 compared with all other weeks (*P* < 0.03 for all; Fig. 2C). Measured EE at week 24 was not different from any other weeks (*P* > 0.05 for all). At a relative workload, measured EE was lower in weeks 1 to 8 than in weeks 12 to 24 (Fig. 2D). A RACE&SEX–time interaction existed for relative workload exercise (*P* < 0.01), but not absolute (*P* = 0.54), with White males having greater measured EE than females across the entire exercise intervention, and Black females having higher measured EE than White females at weeks 12, 16, and 20.

An effect of time was found for RER at an absolute workload (*P* < 0.01; Fig. 2E) but not relative workload (*P* = 0.08; Fig. 2F), with RER increasing after week 1 and a greater RER found at week 20 compared with weeks 6 and 8. A RACE&SEX–time interaction was not found for RER at an absolute or relative workload (*P* = 0.11 for both).

A decrease in V̇O_2_ (mL·kg^−1^·min^−1^) was seen at an absolute workload from weeks 1 to 2, with further reduction occurring by week 24 (time effect; *P* < 0.01; Fig. 2G). Conversely, an increase in V̇O_2_ was seen at relative workload exercise, in combination with increasing speed and/or grade, throughout the intervention (*P* < 0.01; Fig. 2H). Increased V̇O_2_ was seen at week 1 compared with 2, weeks 2 to 4, weeks 4 to 8, weeks 8 to 12, and weeks 16 to 24. In addition, a RACE&SEX effect was found for both exercise workloads, with Black females having lower V̇O_2_ than White participants at an absolute workload and White males having higher V̇O_2_ than female participants at a relative workload (*P* < 0.01 for both). A RACE&SEX–time interaction was found for relative workload exercise (*P* = 0.01), but not absolute (*P* = 0.28), with White males having higher V̇O_2_ than female participants from week 2 and throughout the intervention.

Absolute EOF decreased over the course of the intervention (time effect; *P* < 0.01; Fig. 2I). Furthermore, a RACE&SEX effect was found due to White males having higher absolute EOFs than females and EE being consistently overestimated in Black females compared with White participants (RACE&SEX effect; *P* < 0.01). Relative EOF also decreased over the course of the intervention, with weeks 6, 8, 16, 20, and 24 being significantly lower than week 1 (time effect; *P* = 0.01; Fig. 2J). No RACE&SEX effect was found for relative EOF (*P* = 0.43), and no RACE&SEX–time interaction was found for absolute or relative EOF (*P* = 0.61 and *P* = 0.23, respectively).

#### Adjusting for discrepancies between measured and estimated RMR improved EOF values at a relative and maximal workload, but not absolute

**FIGURE 3:**
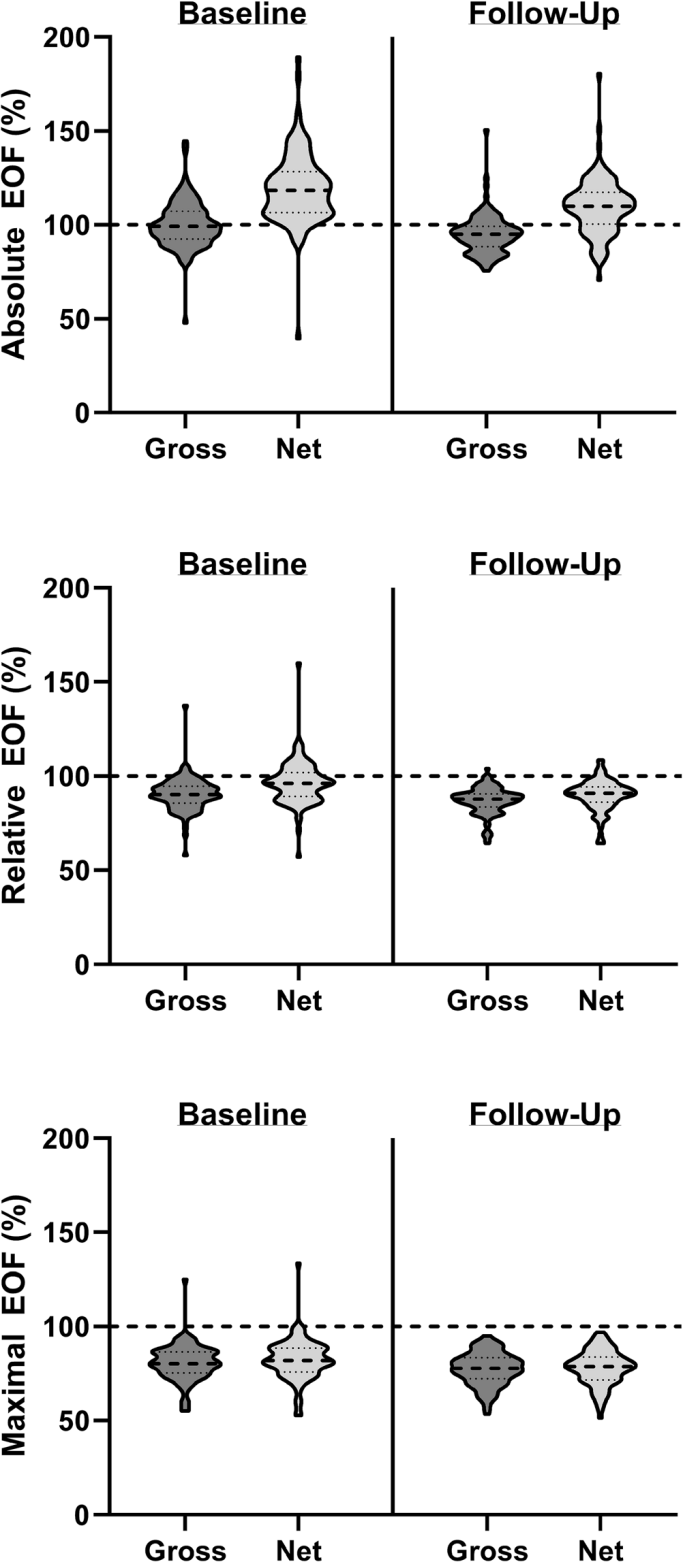
Gross and net EOF at an absolute workload (2.0 mph, 0% grade), relative workload (65%–85% V̇O_2peak_), and maximal workload (maximum EE achieved during graded V̇O_2peak_ test). EOF represents the accuracy of EE estimation equations by expressing measured EE (via indirect calorimetry) as a percent of estimated EE (calculated using the ACSM’s equation for treadmill walking). Net EOF was calculated by removing the resting components from measured and estimated EE.

Because of the discrepancy between measured and estimated RMR, resting V̇O_2_ was investigated for its role in influencing total EE during exercise (Fig. 3). Mean V̇O_2_ at rest increased from 2.54 ± 0.08 to 2.66 ± 0.08 mL⋅kg^−1^⋅min^−1^ from baseline to follow-up (*P* < 0.01). Net EE was calculated by removing the resting component of EE from the measured and estimated gross EE, and a net EOF was calculated for absolute, relative, and maximal workload at baseline and follow-up.

Net EOF was greater, demonstrating an underestimation from ACSM metabolic equation compared with gross EOF at an absolute workload at baseline (118.5% ± 3.8 % vs 99.0% ± 3.8 %, *P* < 0.01). After the 24-wk intervention, net EOF displayed greater accuracy than baseline values (108.6% ± 3.8 % vs 118.5% ± 3.8 %, *P* < 0.01). However, net values remained less accurate than gross values (94.1 ± 3.8 %, *P* < 0.01).

At a relative workload, net EOF represented more accurate estimations of EE than gross values at baseline (96.4% ± 2.3 % vs 90.4% ± 2.3 %, *P* < 0.01). After the 24-wk intervention, both net and gross EOF demonstrated a greater magnitude of overestimation compared with baseline (*P* < 0.01 for both). However, net EOF remained more accurate than gross values (89.8% ± 2.3 % vs 86.8% ± 2.3 %, *P* < 0.01).

Finally, maximal workload exercise elicited the greatest degree of overestimation, with adjustment for RMR improving EOF compared with gross values at baseline (82.4% ± 2.3% ± 80.7% ± 2.7 %, *P* < 0.01) and follow-up (78.2% ± 2.3 % vs 77.5% ± 2.3 %, *P* < 0.01).

## DISCUSSION

This secondary analysis of the E-MECHANIC study aimed to evaluate the accuracy of the ACSM metabolic equation for walking in individuals with overweight and obesity and identify potential modulators for their accuracy across a 24-wk aerobic exercise intervention. Because the original ACSM metabolic equations were derived from data collected in three exercise trained males (age: 23 – 43 yr; weight: 59–77 kg) (12) and 2 male athletes (age: 28-29 yr; weight: 67.5 – 70.0 kg) (13), it was hypothesized that race, sex, and anthropometric variables (including weight, body fat percent, and lean mass) would act as modulators for the metabolic equation. It was additionally hypothesized that exercise training status would act as a modulator for the metabolic equation, and thus, estimation accuracy would improve over the duration of the exercise intervention as participants became more trained. Notably, although race, sex, and anthropometric variables were identified as potential modulators of equation accuracy, we also observed that exercise training led to poorer estimation of EE, perhaps due to improvements in exercise efficiency that exceeded body composition and weight loss-induced changes to EE.

In accordance with previously published literature (16–18), this study showed that the ACSM metabolic equation significantly overestimates EE. The largest relative discrepancy in estimated versus measured EE in untrained adults with overweight and obesity was observed at rest (30.4% lower EE than estimated, equivalent to 699 kcal·d^−1^). EE estimation accuracy decreased with increasing exercise workload (0.44%, 9.2%, 20.3% lower EE than estimated for absolute, relative, and maximal workload exercise, respectively). Similarly, previous research has found a greater degree of error in the ACSM metabolic equation in females with overweight compared with those with a healthy weight and more inaccuracy in jogging (84 ± 29 kJ overestimation at 5 mph) versus walking the same distance (12 ± 25 kJ overestimation at 3 mph for 1 mile) (18). Furthermore, in trained males, the time to achieve an EE goal (400 kcal) at 60% oxygen uptake reserve (V̇O_2_R) was not different when measured compared with when estimated with the ACSM metabolic equation, but it was significantly longer than predicted at 80% V̇O_2_R (27). When the time taken to achieve the 400 kcal EE goal was divided into quartiles, the participants did reach the predicted EE at 80% V̇O_2_R by the last quartile; however, this may be reflective of cardiovascular drift (27). Given the speed limits in the walking equation, a decrease in the difference between walking and running may partially explain the increasing inaccuracy with increasing workload and reflect the limits of the equation. Therefore, the present study and previous evidence suggest that ACSM metabolic equation accuracy may be reduced in individuals with overweight or obesity and at higher exercise workloads.

Improvements in exercise efficiency, identified via decreased measured EE at an absolute workload, were found within 2 wk of starting the exercise intervention, with minimal further benefits to efficiency after 8 wk of training. Published literature has identified training status as a modulator for exercise efficiency (28–30); however, this study provides novel insight into the time course over which adaptations may occur in previously sedentary individuals by measuring EE at weeks 1, 2, 4, 6, 8, 12, 16, 20, and 24 of the exercise intervention. After 6 months of aerobic exercise training, Woo and colleagues (28) found a 17% improvement in exercise efficiency during treadmill walking at 3.5 mph and 0% grade. Furthermore, older adults who run for at least 30 min, 3 times per week for at least 6 months have 7%–10% reductions in energy consumption during treadmill walking compared with older adults who spend the same time walking, despite no differences in walking biomechanics (29). Although we are not able to identify the contributions of metabolic and mechanical adaptations to the improvement in exercise efficiency in this study, exercise efficiency in older adults has been correlated with muscle health and mitochondrial capacity (31). In addition, we have shown that reductions in EE at an absolute workload may occur within 2 wk.

Race and sex were identified as key modulators for ACSM metabolic equation accuracy, with a consistently greater degree of overestimation in Black females than White males at an absolute workload. Following the exercise intervention, Black females were expending 29 kcal less than estimated per hour of walking (2 mph and 0% grade). In addition, following the ACSM guidelines for general health (150 min of moderate-intensity exercise per week) would result in an overestimation of 209 kcal·wk^−1^ in Black females. In the context of weight loss studies lasting many months, this degree of inaccuracy could accumulate and result in lower-than-expected weight loss. Across a year, this could translate to losing 3.1 lb less than predicted. Age, V̇O_2peak_, and anthropometric variables were also related to metabolic equation accuracy; however, the contributions of these variables to explaining variation in EOF were inconsistent among exercise workloads. Therefore, based on the current data, it is unclear which measures may be the best addition to future revisions of metabolic equations to improve accuracy. Fat mass was related to baseline EOF at all workloads. Because of the lower metabolic activity of adipose tissue compared with skeletal muscle, excess adiposity is likely a predominant factor in the problems associated with EE estimation assumptions in individuals with obesity, particularly females (32). In addition, a relationship between truncal fat-free mass and RMR has been identified, specifically with African Americans having lower RMR and smaller metabolically active organs within the truncal region than non-Hispanic Caucasians. Consequently, the detrimental effects of adiposity on EE estimation accuracy may compound race-related differences in high-metabolic rate organ mass (33,34), resulting in greater differences between races at lower intensities where the metabolic activity of intra-abdominal organs has a greater contribution to total EE. In the absence of accurate body composition measures, adding physiological variables including gender, age, BMI, and physical activity levels increases the coefficient of determination (*R*^2^) of predictive models of V̇O_2max_ by 0.2 (35), and therefore, may lead to better prediction of EE at maximal intensity. Evidence from the present study supports the addition of these physiological variables, following further investigation into the optimal combination of measures for accuracy and practical application.

Furthermore, the relative contributions of V̇O_2_ and RER to EE differ by race and sex. At relative workloads, Black females had higher RER than White participants, and females had lower V̇O_2_ than White males. These findings are similar in females with a healthy weight, in which RER was higher in African American females during exercise (30-min treadmill exercise at 65% V̇O_2max_) and at rest compared with Caucasian females (36). In addition, V̇O_2_ was lower at rest in African American versus Caucasian females (36), and healthy African American males had lower fat oxidation than Caucasian males, which may have important implications for the development of obesity and associated diseases (37). The application of these findings may be limited to untrained or individuals who are not normal weight because previous research in trained adults has suggested that the RER is lower in females than males during exercise (38). As a result, substrate utilization may have been influenced by confounding variables, including energy and glycogen availability, which are potentially less relevant to sedentary individuals with overweight or obesity. Specifically, in individuals with overweight or obesity, we provide evidence to support a higher RER in Black females compared with White males and females.

A strength of this study is that all exercise sessions were supervised, and EE during training was closely monitored to allow more accurate compliance to exercise dose. A limitation of this study is the low sample size (*n* = 4) for Black males. Consequently, it was not possible to distinguish between race effects and sex effects, because the groups could be skewed by the lack of Black males and may not provide an accurate representation of the proposed analysis. In addition, using an estimated standard caloric equivalent (5.0 kcal·L^−1^ O_2_), as suggested in the ACSM guidelines, to estimate EE from the metabolic equations may have resulted in an increase in the inaccuracy of the equation. Given that the maximum range of substrate oxidation is from 4.686 to 5.047 kcal·L^−1^ O_2_ (7), the maximum possible error from substrate oxidation estimates is approximately 6%; however, exercising during the testing increased RER closer to 5.0 kcal·L^−1^ O_2_ than 4.686 kcal·L^−1^ O_2_, thereby reducing the maximum possible error caused by this assumption and failing to account for the entirety of the inaccuracies previously reported. Furthermore, during the V̇O_2peak_ test, 2-min stages were used, which may have been insufficient time for participants to reach steady state and for the metabolic cart to detect increases in oxygen consumption.

## CONCLUSIONS

In summary, we observed that the ACSM metabolic equation for walking overestimates EE in sedentary individuals with overweight or obesity. During an exercise bout, the accuracy of the metabolic equation decreases with increasing exercise workload. In addition, race, sex, age, fat mass, and V̇O_2peak_ were all identified as modulators for metabolic equation accuracy. Accordingly, Black females may experience greater overestimation of EE than White individuals, particularly at lower exercise workloads. These findings may partly explain the observed differences in body weight changes in response to exercise interventions that employed ACSM metabolic equations to set EE prescriptions. Future studies might consider modifying the ACSM metabolic equations in efforts to improve the accuracy of EE estimations, particularly in diverse populations. Thus, careful consideration is needed when using the metabolic equation for walking to prescribe exercise dose, particularly in weight loss studies, to better account for precise caloric expenditure and adaptations improving exercise efficiency.
